# Chromatographic evaluation of tocols and sterols of processed canola oil and deodorizer distillate

**DOI:** 10.55730/1300-0527.3307

**Published:** 2021-09-12

**Authors:** Hadia SHOAIB, Syed Tufail Hussain SHERAZI, Saba NAZ, Sarfaraz Ahmed MAHESAR, Abdul Rauf KHASKHELI, Siraj UDDIN, Ahmed Raza SIDHU, Hamide Filiz AYYILDIZ, Hüseyin KARA, Mustafa TOPKAFA

**Affiliations:** 1 National Centre of Excellence in Analytical Chemistry, University of Sindh, Jamshoro, Pakistan; 2 Dr. M.A Kazi Institute of Chemistry, University of Sindh, Jamshoro, Pakistan; 3 Institute of Pharmacy, Shaheed Mohtarma Benazir Bhutto Medical University, Sindh, Pakistan; 4 International Center for Chemical and Biological Sciences, HEJ Research Institute of Chemistry, University of Karachi, Karachi, Pakistan; 5 Department of Basic Pharmaceutical Sciences, Faculty of Pharmacy, Selçuk University Konya, Turkey; 6 Faculty of Science, Department of Chemistry, Selçuk University, Konya, Turkey; 7 Vocational School of Technical Sciences, Department of Chemistry and Chemical Processing Technologies, Konya Technical University, Turkey

**Keywords:** Canola oil, deodorizer distillate, processing, tocols, sterols

## Abstract

Tocopherols and tocotrienols in the combined form are known as tocols. Changes of total and individual tocols and sterols concentration of canola oil and deodorizer distillate (DD) during different processing stages were evaluated with the application of gas chromatography (GC) and high-performance liquid chromatography (HPLC). For sterols analysis, GC coupled with flame ionization detector (FID) was used while tocols in canola oil samples and DD, normal phase (NP) HPLC was applied. The results of the present study indicated that levels of total and individual tocols and sterols content were decreased during processing (neutralization to deodorization). Deodorization was found to be the most effective process for the reduction of total sterols and tocols as 55.9% and 34.2%, respectively. A high amount of tocols and sterols was observed in DD. Among tocols and sterols; beta tocopherol (β-T) and β-sitosterol were found to be in greater concentration 53.97% and 31.82%, respectively. Therefore, DD could be used as a valuable by-product in the cosmetics and food industries.

## 1. Introduction

Canola oil is extracted from rapeseeds, which is a bright yellow flower of the family Brassicaceae. It is an essential oil crop mainly grown in United States, Canada, China, Australia, India, and the European Union. In recent years, the cultivation of canola crops has significantly increased day by day [1]. In the production of oil, the quality of the oil is the main factor because it decides the approval and sales of the products which are derived from vegetable oil [2, 3]. On the other hand, the fatty acid composition and its minor components, for instance, free fatty acids (FFA), color pigments, trace metals, phenolic compounds, waxes, and phospholipids of vegetable oil often affect the stability and quality of oil [4]. For that reason, various efficient industrial processing stages are used for removing these disagreeable impurities with the minimum effect on the desired components and the low possible loss of neutral oil.

The main steps of vegetable oil refining include neutralization, bleaching, and deodorization. However, refining can also cause the removal of desirable health-promoting minor components from the oil [5]. In the neutralization process, NaOH is used to eliminate the FFA level from the oil; however, in the bleaching process, bleaching clay is used to absorb the color pigments and trace metals. Deodorization is the final key step of the refining process accountable for removing targeted volatile compounds that are liable for producing unacceptable odor, color, taste, and flavor in the oil. Unfortunately, these processing stages also result in the reduction of very important bioactive components such as tocols, sterols, phenols, and aromas. The level of reduction of each bioactive component depends on processing parameters, quality, and nature of the input oil [6]. However, a significant amount of phytosterols and tocols are distilled and recovered as by-products in the form of DD, which has been considered to be a rich source of these bioactive components. It is a by-product of the vegetable oil deodorization process and is a complex mixture of FFA, glyceride, tocols, phytosterol (free and esterified), hydrocarbon, and other volatile molecules such as aldehyde, ketone, and peroxide [7–9]. Tocols are the natural antioxidants that are known as “Vitamin E”. It is a naturally occurring antioxidant, found in most of the oilseeds that are extracted during the refining of oil. It has different antioxidants and biological abilities, which helps to decrease the low-density lipoprotein (LDL) in biological membranes, prevent lipid from oxidation, terminate the free radical chain reactions, and additionally increases the stability of the vegetable oil [10]. There are four homologs within the tocopherol groups: alpha (α), beta (β), gamma (γ), and delta (δ), which differ in their antioxidant activities [11, 12]. α-tocopherol has high in *vivo* biological activity, whereas γ-tocopherol has utmost activity in food lipids [13]. Subsequently, foods which contain tocols are very beneficial to human health and promote the stability of food products [14]. Sterols are found widely in plants, animals, and fungi, and are made up of three cyclohexane rings, one cyclopentane ring, and an alcohol group. They play a vital role as structural components in cell membranes because they perform signal transduction, control the activity of membrane-bound enzymes, and regulate membranes. Sterols occur as phytosterols in plants and are most widely known for their LDL cholesterol-lowering properties [15–18]. Tocols, as well as sterols, are antioxidants and their investigation in the oils and fats is very important to know their value and applications. Also, phytosterols are useful bioactive compounds in pharmaceuticals for the production of therapeutic steroids, anticancer medicines, preparation of cosmetics, and also used as additives in functional foods. The present study aimed to use GC and HPLC techniques to evaluate the impact of overall and individual processing stages such as neutralization, bleaching, and deodorization on the reduction of tocols and sterols of canola oil and its DD.

## 2. Materials and methods

### 2.1. Reagents and sample collection

All the chemicals and reagents including standards of tocols such as alpha-, beta-, gamma-, and delta-tocopherols (α-T, β-T, γ-T, δ-T), tocotrienols (α-TT, β-TT, γ-TT, δ-TT), and sterols (cholesterol, campesterol, stigmasterol, β-sitosterol, and avenasterol) were purchased from E-Merck (Darmstadt, Germany). Samples of crude, neutralized, bleached, and deodorized processed canola oils, as well as DD from the same batch, were obtained from an industry located in Karachi, Pakistan. All samples were stored in amber glass bottles purged with nitrogen gas to avoid oxidation and stored at −4 °C until they were analyzed.

### 2.2. Examination of tocols in canola oil samples and DD by NP-HPLC

Official AOCS method Ce 8–89 was used for the separation of tocols in canola oil samples and DD [19]. Tocols composition was determined by using NP-HPLC (Agilent 1200 series) system fitted with a fluorescence detector (FLD) (Agilent Technologies Inc., Wilmington, DE, USA). Chemstation B.03.02–2008 data processor was used for the separation of tocols. For the preparation of canola oil samples of DD for tocols analysis, about 1 g of sample was taken and mixed with 10 mL hexane. About 20 μL of this mixture was injected into the LiChrospher Si 100–5 column (250 × 4 mm, 5 μm film thickness, Hichrom, England). A mobile phase mixture (0.4:99.6, v/v) hexane and iso-propanol were used at a flow rate of 1 mL min^−1^ with isocratic elution. For excitation, the wavelength of FLD was set 290 nm and for emission, the wavelength of FLD was set at 330 nm. Tocols peaks (or peak heights) in oils were identified by reference to the chromatograms obtained from standards and recorded the areas under the peak and quantified results were reported as mg/kg.

### 2.3. Determination of sterols composition by GC-FID

For sterols analysis, the unsaponifiable matters of all canola oil samples and DD were extracted by the official AOCS method Ca 6a-40 [19]. After extraction, the unsaponifiable matters were dissolved in 5 mL of hexane. Then the sample solution was run on the GC-FID instrument (Agilent 7890 series) used for sterols analysis. For the separation of sterols, the HP-88 column (100 m, 0.25mm Agilent Technologies) was used. About 2 μL of an aliquot was injected in a splitless mode. As a carrier gas, helium (He) was used with a flow rate of 10.2 mL/min. The initial temperature programming of the oven was set to 100 °C and then increased to 10 °C /min to the final temperature of 295 °C and hold for 20 min. For the confirmation of the sterol peaks, the retention times of the authentic standards were compared. The peak areas under each sterol were determined as relative peak areas to the total peak area of all sterols.

### 2.4. Statistical analysis

Identification of tocols, and sterols in crude, neutralized, bleached, deodorized canola oils and DD samples was carried out based on retention times of standards. Statistical analysis of the data was carried out using Minitab 16 USA software. Data were analyzed by analysis of variance (ANOVA) followed by the Tukey test (p ≤ 0.05). Results are reported as mean ± (SD) of three replicates (each replicate corresponds to a different batch of refining).

## 3. Results and discussion

### 3.1. Tocols composition of crude and industrially processed canola oil

**Figure 1:**
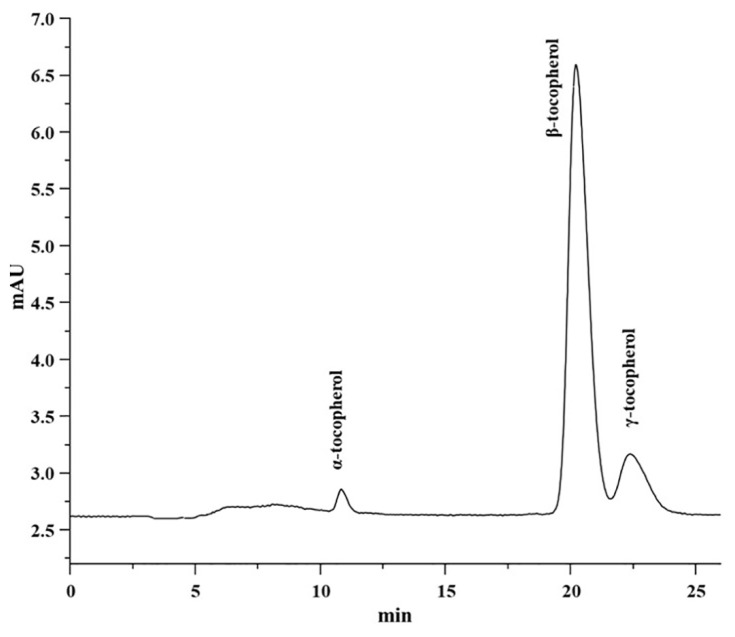
HPLC representative chromatogram of tocols of refined canola oil

Table 1 displayed the results of HPLC analysis of the tocols profile of (crude to deodorized) canola oil samples such as α-tocopherol, β-tocopherol, and γ-tocopherol. Figure 1 shows the representative chromatogram of tocols of refined canola oil. According to NP-HPLC results, the β-tocopherol presents in higher quantities in crude compared to deodorized canola oil. While α-tocopherol was found to be the second most abundant tocopherol present in crude, neutralized, bleached, and deodorized oil. The lowest concentration of γ-tocopherol was determined in crude and industrially processed canola oils. In the current study, levels of α-, β-, and γ-tocopherols in crude canola oil were found to be 90.32, 260.16, and 60.60 mg/kg, respectively. Ghazani et al. [20] reported only two tocols in canola oil. The levels of α-tocopherol (154.1 mg/kg) and γ-tocopherol (338.4 mg/kg) were higher in crude oil as compared to the results of the present study. In neutralized oil, the amounts α-, β-, and γ-tocopherols were determined as 80.83, 230.83, and 60.60 mg/kg, respectively. Ghazani et al. [20] reported a higher concentration of α-tocopherols (107.3 mg/kg) and γ-tocopherol (244.1 mg/kg) as compared to current results. In bleached oil, α-, β-, and γ-tocopherols were found as 40.62, 170.01, and 50.59 mg/kg, respectively. The level of α-tocopherol (103.1 mg/kg) and γ-tocopherol (287.5 mg/kg) were reported higher by Ghazani et al. [20]. In the deodorized oil, α-, β-, and γ-tocopherols were further reduced to 30.75, 120.11, and 20.90 mg/kg, respectively. The results of deodorized oil for tocopherols separation, detection, and quantification were also compared with Ayyildiz et al. and Ghazani et al. [16, 20].

However, the level of α- and γ-tocopherols after the deodorization process were found significantly lower while β-tocopherol was considerably higher than the studies reported by Ayyildiz et al. and Ghazani et al. [16, 20]. Different concentrations of tocols present in crude and processed oils reported by different researchers may be due to the different variety of canola seed, diverse geographical and environmental conditions as well as different extraction and processing parameters. Also, the efficiency of the applied method is very important to separate the peaks of individual tocols and quantification at a lower concentration. No δ-tocopherols and tocotrienols were detected in crude, neutralized, bleached, and deodorized canola oil samples.

### 3.2. Impact of processing on tocols composition of crude and industrially processed canola oil

During the neutralization process, α-, β-, and γ-tocopherols were reduced from crude to neutralized oil. The impact of neutralization on the reduction of α-, β-, and γ-tocopherols was found to be 10.5%, 11.27%, and 0.03%, respectively as shown in Table 2. In the current study, the influence of neutralization on α- and γ-tocopherols was found to be lower than 30.4% and 27.4% reported by Ghazani et al. [20]. This may be due to the different processing conditions or different concentrations of these tocols in their respective crude canola oils. In the bleaching process, α-, β-, and γ-tocopherols were reduced from neutralized to bleached oil. The impact of bleaching on the reduction of α-, β-, and γ-tocopherols was found to be 49.75%, 26.35%, and 16.52%, respectively. In our study, the impact of bleaching on α- and γ-tocopherols was found to be higher than that reported by Ghazani et al. [20].

During the deodorization process, α-, β-, and γ-tocopherols were reduced from bleached to deodorized oil. The impact of deodorization on the reduction of α-, β-, and γ-tocopherols was found to be 24.29%, 29.35%, and 58.69%, respectively. In the present study, the impact of deodorization on α- and γ-tocopherols was found to be higher than 9.7% and 18.7% reported by Ghazani et al. [20]. In the current study, the impact of neutralization on the loss of total tocols content was found to be 9.44%, which was lower than 19.6% reported by Ghazani et al. [20]. In the bleaching and deodorization processes, the impact on the reduction of total tocols was found to be 29.8% and 34.2%, respectively, which was higher than 2.6% and 16.3% reported by Ghazani et al. [20].

The overall impact of processing on the reduction of α-, β-, and γ-tocopherols during neutralization, bleaching, and deodorization was found to be 65.95%, 53.83%, and 65.51%, respectively. In the current study, the impact of processing on α-, β-, and γ-tocopherols was found to be higher than 39.6% and 30.9% reported by Ghazani et al. [20]. Correspondingly, the overall impact of processing on the total tocols content from crude to deodorized canola oil was found to be 58.2% which was also higher than 33.6% reported by Ghazani et al. [20].

### 3.3. Tocols composition of DD of canola oil

Table 3 shows the tocols composition of canola oil DD. Among all tocols, the β-tocopherol was present in higher quantity while δ-tocopherol was found to be lower in DD of canola oil. In the current study, levels of α-, β-, γ-, and δ-tocopherols in DD were found to be 17.82%, 53.97%, 12.35%, and 6.76%, respectively, while only one tocotrienol, i.e. α-tocotrienols was observed as 9.11% in DD. It was not detected in crude, neutralized, bleached, and deodorized oil. Maybe the reason is that it is present in small amounts in these oils but when these trace amounts are distilled and collected in DD in the concentrated form then their presence was confirmed by HPLC. Ramamurthi and McCurdy reported two tocopherols, i.e. α- and γ-tocopherols. The relative percentages of α- and γ-tocopherols (24% and 65%, respectively) were higher as compared to the results of the present study [22]. Durant et al. reported three tocopherols: α-, γ-, and δ-tocopherols. The levels of relative percentages of γ and δ–tocopherols (65.52% and 31.81%) were higher while the level of α-tocopherol was lower as compared to the results of the present study [22]. Naz et al. reported two tocopherols, i.e. α- and γ-tocopherols. The levels of relative percentages of α- and γ-tocopherols (52.35% and 47.65%) were higher as compared to the results of the present study [24].

### 3.4. Sterol composition of crude and industrially processed canola oil

**Figure 2:**
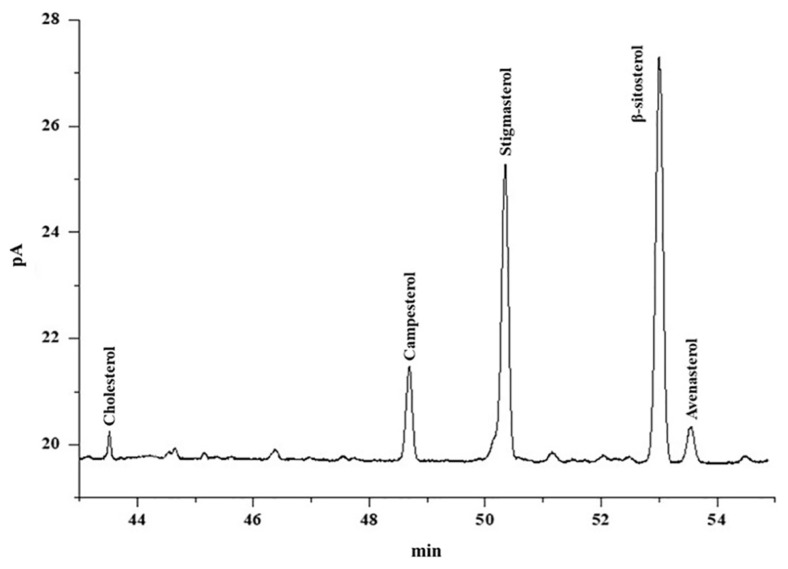
GC-FID representative chromatogram of sterols of refined canola oil

The composition of sterol was evaluated in crude to deodorized canola oil by using GC-FID. Figure 2 is the representative chromatogram of sterols of refined canola oil. Table 4 shows the composition of sterols in the unsaponifiable portion of crude, neutralized, bleached, and deodorized canola oil. The unsaponifiable extracts of crude, neutralized, bleached, and deodorized canola oil samples were used to check sterols profile containing cholesterol, campesterol, stigmasterol, β-sitosterol, and avenasterol. Among the sterols, β-sitosterol was found dominant while cholesterol was determined in the least quantity in crude to deodorized canola oil. In the current study, the level of cholesterol, campesterol, stigmasterol, β-sitosterol, and avenasterol in crude canola oil was found to be 3.17, 11.8, 41.1, 54.32, and 6.10 mg/kg, respectively.

The results of the present study in crude to deodorized canola oil were compared with those reported by Ghazani et al. and Özcan et al. [20, 21]. The results of the present study in crude to deodorized canola oil were lower than the reported study by Özcan et al. [21] while some results of the present study are lower and higher than those reported by Ghazani et al. [20]. The levels of campesterol (324.4 mg/kg) and β-sitosterol (491.9 mg/kg) were higher, whereas the level of stigmasterol (10.6 mg/kg) was lower in crude oil as compared to the results of the present study [20]. In neutralized oil, the amounts of cholesterol, campesterol, stigmasterol, β-sitosterol, and avenasterol were determined as 3.11, 11.3, 39.6, 52.3, and 6.3 mg/kg, respectively. Ghazani et al. reported a higher concentration of campesterol (301.8 mg/kg) and β-sitosterol (445.3 mg/kg) and a lower concentration of stigmasterol (5.8 mg/kg) as compared to current results [20]. In bleached oil, cholesterol, campesterol, stigmasterol, β-sitosterol, and avenasterol were found 3.07, 9.7, 35.2, 48.4, and 5.97 mg/kg, respectively. The levels of campesterol (316.2 mg/kg) and β-sitosterol (471.7 mg/kg) were greater, while stigmasterol (6.4 mg/kg) was lower than that reported by Ghazani et al. [20]. In the deodorized oil, the cholesterol, campesterol, stigmasterol, β-sitosterol, and avenasterol were further reduced to 2.87, 4.2, 13.9, 19.8, and 4.4 mg/kg, respectively. In our present study campesterol and β-sitosterol were found to be higher than 275.0 and 439.8 mg/kg in the reported study, while stigmasterol was found to be lower than 3.2 mg/kg in th reported study [20]. Different concentrations of sterols in crude and processed oils have been reported by many researchers. This variation may be due to the different varieties of canola seed, diverse geographical and environmental conditions as well as extraction and processing parameters.

### 3.5. Impact of processing on sterols composition of crude and industrially processed canola oil

Table 5 shows the impact of industrial processing on the sterols composition of crude and processed canola oils. During the neutralization process, cholesterol, campesterol, stigmasterol, β-sitosterol, and avenasterol were reduced from crude to neutralized oil. The impact of neutralization on cholesterol, campesterol, stigmasterol, β-sitosterol, and avenasterol was found to be 1.89%, 4.24%, 3.65%, 3.68%, and 3.28%, respectively. In our study, the impact of neutralization on the reduction of campesterol, stigmasterol, and β-sitosterol was found to be lower than 6.97%, 45.28%, and 9.47% reported in [20]. In the bleaching process, cholesterol, campesterol, stigmasterol, β-sitosterol, and avenasterol were reduced from neutralized to bleached oil. The impact of bleaching on cholesterol, campesterol, stigmasterol, β-sitosterol, and avenasterol was found to be 1.29%, 14.16%, 11.11%, 7.46%, and6.35%, respectively. In the reported study, from neutralized to bleached oil, the levels of campesterol, stigmasterol, and β-sitosterol were increased and the impact of bleaching on campesterol was found to be higher at 4.77%, whereas that on stigmasterol and β-sitosterol was found to be similar (10.34% and 6.77%) [20]. During the deodorization process, cholesterol, campesterol, stigmasterol, β-sitosterol, and avenasterol were reduced from bleached to deodorized oil. The impact of deodorization on cholesterol, campesterol, stigmasterol, β-sitosterol, and avenasterol was found to be 6.51%, 56.70%, 60.51%, 59.09%, and 25.29%, respectively. In the current study, the impact of deodorization on the levels of campesterol, stigmasterol, and β-sitosterol was found to be higher than 13.02%, 50%, and 6.77% reported in [20]. In the present study, the impact of neutralization on the loss of total sterols content was found to be 3.33% which was lower than that of the reported study (9.70%). In the bleaching and deodorization process, the impact on loss of total sterols content was found to be 9.12% and 55.86%, respectively which was higher (6.02% and 9.66%) than that reported by Ghazani et al. [20].

During processing, cholesterol, campesterol, stigmasterol, β-sitosterol, and avenasterol were reduced from crude to deodorized oil. The overall impact of processing on cholesterol, campesterol, stigmasterol, β-sitosterol, and avenasterol was found to be 9.43%, 64.41%, 66.18%, 63.55%, and 27.87%, respectively. In the current study, the impact of processing on campesterol and β-sitosterol was found to be higher, while stigmasterol was lower than that in the reported study (15.23%, 69.81%, and 10.59%) [20]. The overall impact of processing on the reduction of total sterols content from crude to deodorized canola oil was found to 61.22%, which was higher (13.52%) than that reported by Ghazani et al. [20].

### 3.6. Sterol composition of DD of canola oil

**Figure 3:**
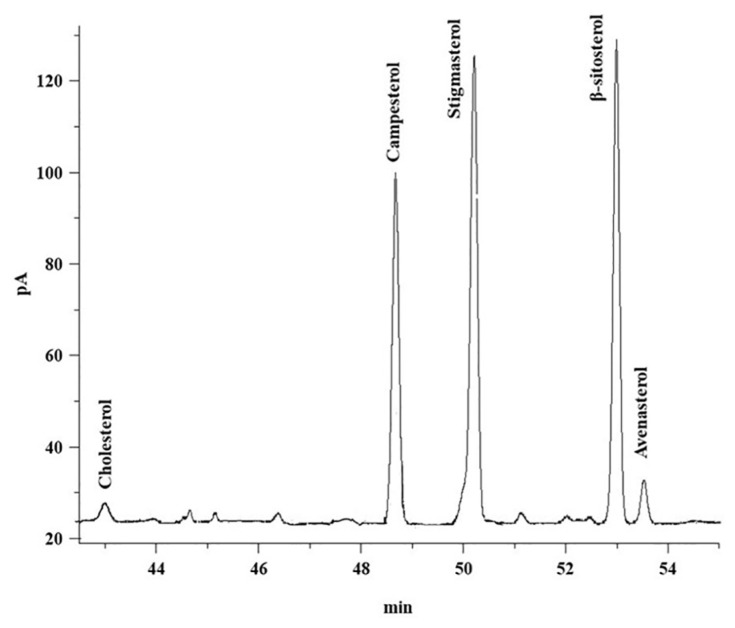
GC-FID representative chromatogram of sterols composition of DD of canola oil

Table 6 shows the sterol composition of unsaponifiable extracts of canola oil DD. Figure 3 shows the representative chromatogram of sterols composition of DD of canola oil. Among all sterols, β-sitosterol was found to be higher, while cholesterol was found to be lower in the DD of canola oil.

In the current study, levels of cholesterol, campesterol, stigmasterol, β-sitosterol, and avenasterol in DD were found to be 5.68%, 23.86%, 29.54%, 31.82%, and 9.09%, respectively. Ramamurthi and McCurdy (1993) reported campesterol and β-sitosterol in DD of canola oil and their relative percentages (29.73% and 70.27%, respectively) higher as compared to the results of the present study [22]. In another study, Durant et al. found campesterol and β-sitosterol in DD of canola oil. The relative percentages of campesterol (82.18%) and β-sitosterol (17.82%) were reported higher as compared to the results of the present study [23]. Naz et al. reported three sterols including campesterol, stigmasterol, and β-sitosterol in the DD of canola oil [24]. The relative percentages of campesterol (31.48%) and β-sitosterol (57.30%) were higher, while stigmasterol (11.21%) was lower as compared to the results of the present study.

## 4. Conclusion

The results of the present study indicated that overall industrial processing such as neutralization, bleaching, and deodorization was found to be responsible for the reduction of tocols and sterols which means that the nutrition value and stability of canola oil are compromised. However, these useful components are collected in the form of waste by-product (DD) which is the richest source of tocols/sterols and could find potential applications in the food and cosmetics industries. The work on utilization of DD is on the way but at the cost of edible oil nutritive efficiency and consumer health, which is not agreeable. Therefore, there is a strong need to improve the processing conditions in which there should be no loss or minimum loss of these valuable components in edible oil and increase consumer acceptance towards natural health products.

## Figures and Tables

**Table 1:** Tocol’s composition of crude and industrially processed canola oil.

Tocols (mg/kg)	Crude oil	Neutralized oil	Bleached oil	Deodorized oil
α-T	90.3 ± 0.20^a^	80.8 ± 0.55^b^	40.6 ± 0.82^c^	30.8 ± 3.02^d^
β-T	260.2 ± 0.62^a^	230.8 ± 0.61^b^	170.0 ± 0.82^c^	120.1 ± 0.15^d^
γ-T	60.6 ± 1.95^a^	60.60 ± 1.87^b^	50.6 ± 2.01^c^	20.9 ± 1.64^d^
Total	411.1	372.3	261.2	171.7

T = Tocopherols. The values provided in the table are the mean values of triplicate analysis with standard deviation,

a–d different letters indicate a significant difference of tocols among industrial processes at p <0.05.

**Table 2:** Impact on Tocol’s composition of crude and industrially processed canola oil.

Tocols (%)	C-N	N-B	B-D	C-D
α-T	10.5	49.6	24.29	65.95
β-T	11.27	26.3	29.35	53.83
γ-T	0.03	16.52	58.69	65.51
Total reduction	9.44	29.8	34.2	58.2

T= Tocopherols, C-N= Crude to neutralized, N-B = Neutralized to bleached, B-D = Bleached to deodorized, C-D = Crude to deodorized

Impact of neutralization (%) = Difference of crude to neutralized/crude×100

Impact of bleaching (%) = Difference of neutralized to bleached/neutralized×100

Impact of bleaching (%) = Difference of bleached to deodorized/bleached×100

Overall/total Impact (%) = Difference of crude to deodorized/crude×100

**Table 3:** Tocol’s composition of DD of canola oil

Tocols (%)	DD	Ramamurthi and McCurdy [22]	Durant et al. [23]	Naz et al. [24]
α-T	17.82 ± 0.76	24	2.67	52.35
α-TT	9.11 ± 0.37	nd	nd	nd
β-T	53.97 ± 1.79	nd	nd	nd
γ-T	12.35 ± 0.60	65	65.52	47.65
δ-T	6.76 ± 0.29	nd	31.81	nd

nd, not detected; T= Tocopherols and TT= Tocotrienols. The values provided in the Table are the mean values of triplicate analysis with standard deviation.

**Table 4:** Sterol’s composition of crude and industrially processed canola oil

Sterols (mg/kg)	Crude oil	Neutralized oil	Bleached oil	Deodorized oil
Cholesterol	3.17 ± 0.15^a^	3.11 ± 0.09^b^	3.07 ± 0.11^c^	2.87 ± 0.20^d^
Campesterol	11.8 ± 0.47^a^	11.3 ± 0.42^b^	9.7 ± 0.31^c^	4.2 ± 0.51^d^
Stigmasterol	41.1 ± 1.77^a^	39.6 ± 1.63^b^	35.2 ± 0.92^c^	13.9 ± 1.81^d^
β-sitosterol	54.3 ± 1.29^a^	52.3 ± 2.18^b^	48.4 ± 1.21^c^	19.8 ± 0.97^d^
Avenasterol	6.10 ± 0.25^a^	6.3 ± 0.31^b^	5.97 ± 0.26^c^	4.4 ± 0.06^d^
Total	116.49	112.61	102.34	45.17

nd, detected; a, crude oil; b, neutralized oil, c, bleached oil; d, deodorized oil. The values provided in the table are the mean values of triplicate analysis with standard deviation,

a–d different letters indicate a significant difference of Sterols among industrial processes at p <0.05.

**Table 5:** Impact of industrial processing on sterols composition of crude and processed canola oils.

Sterol (%)	C-N	N-B	B-D	C-D
Cholesterol	1.89	1.29	6.51	9.43
Campesterol	4.24	14.16	56.70	64.41
Stigmasterol	3.65	11.11	60.51	66.18
Beta Sitosterol	3.68	7.46	59.09	63.55
Avenasterol	3.28	6.35	25.29	27.87
Total reduction	3.33	9.12	55.86	61.22

C-N= Crude to neutralized, N-B= Neutralized to bleached, B-D= Bleached to deodorized, C-D= Crude to deodorized.

Impact of neutralization (%) = Difference of crude to neutralized/crude×100

Impact of bleaching (%) = Difference of neutralized to bleached/neutralized×100

Impact of bleaching (%) = Difference of bleached to deodorized/bleached×100

Overall/total Impact (%) = Difference of crude to deodorized/crude×100

**Table 6:** Sterol’s composition of DD of canola oil.

Sterol (%)	DD	Ramamurthi and McCurdy [22]	Durant et al. [23]	Naz et al. [24]
Cholesterol	5.68 ± 0.24	nd	nd	nd
Campesterol	23.86 ± 1.04	29.73	82.18	31.48
Stigmasterol	29.54 ± 0.22	nd	nd	11.21
β-sitosterol	31.82 ± 1.32	70.27	17.82	57.30
Avenasterol	9.09 ± 0.32	nd	nd	nd

(nd), not detected. The values provided in the table are the mean values of triplicate analysis with standard deviation.
